# Irish traveller women and sexual assault: Patterns of engagement with sexual assault treatment units

**DOI:** 10.1002/ijgo.71022

**Published:** 2026-04-18

**Authors:** Daniel Kane, Aoife McEvoy, Christine Pucillo, Kate O. Halloran, Laura Feely, Nicola Maher, Karen Flood, Maeve Eogan

**Affiliations:** ^1^ Department of Obstetrics and Gynaecology Royal College of Surgeons in Ireland Dublin Ireland; ^2^ Sexual Assault Treatment Unit Rotunda Hospital Dublin Ireland

**Keywords:** forensic examination, rape, sexual assault, sexual health, violence, women's health

## Abstract

**Objective:**

To detail the attendance of female Irish Travellers at the national Sexual Assault Treatment Unit (SATU) network.

**Methods:**

Retrospective cross‐sectional study examining the attendance of female patients who identified as members of the Irish Traveller community at the national SATU network between 2017 and 2023 and comparing them with all other female attenders.

**Results:**

There were 5942 female attendances during the study period, of which 54 were Irish Traveller women with a mean age of 25.9 ± 11.5 years; with 35 (64.8%) presenting within 7 days of assault. Most attended with police involvement (40, 74.1%), assaults most commonly occurred in the victim's home (14, 25.9%) or outdoors (13, 24.1%), and perpetrators were frequently family members (13, 24.1%), intimate/ex‐intimate partners (12 combined, 22.2%), acquaintances of more than 24 h (8, 14.8%), or strangers (9, 16.7%). Compared with other female attenders, Irish Traveller women were significantly less likely to present within 24 h (24.1% vs 39.7%; odds ratio [OR] 0.48 *P* = 0.02) and to report alcohol use in the preceding 24 h (OR 0.33, *P* < 0.001), but were significantly more likely to report a family member (OR 8.25, *P* < 0.001) or an intimate/ex‐intimate partner (OR 2.04, *P* = 0.03) as the assailant.

**Conclusion:**

This study provides the first national analysis of Irish Traveller women attending SATU in Ireland and highlights distinct differences in the patterns of perpetrator relationship and timing of presentation compared with other female attenders.

## 1 INTRODUCTION

Sexual violence is common in Ireland, with one in two Irish women experiencing some form of sexual violence; however, similar to other countries, it remains widely underreported.^1^ For women from marginalized groups, experiences of sexual violence are often further compounded by systemic discrimination, mistrust of services, and barriers to accessing appropriate care. One such marginalized group is Irish Travellers, whose ethnicity was formally recognized by the Republic of Ireland in 2017 following decades of advocacy by Irish Traveller organizations and human rights groups.^2^ According to the 2022 Irish Census, there were 16 172 Irish female Travellers living in the state.^3^

Similar to other Traveller ethnicities, including Roma populations, Irish Travellers experience and describe profound marginalization, discrimination, and social exclusion across Europe and internationally.^4^, ^5^, ^6^ Within this context of systemic disadvantage, Irish Traveller women and girls are at increased risk of gender‐based violence, including sexual assault, intimate partner violence, and domestic abuse.^7^, ^8^, ^9^ Despite this heightened vulnerability, there remains an absence of published research examining the prevalence and characteristics of sexual violence within the Irish Traveller population.

The aim of this paper is to examine attendances at the Sexual Assault Treatment Unit (SATU) network in Ireland among women who identified as Irish Travellers, and to describe the demographic characteristics, incident‐related factors, and attendance details of these women. In doing so, this study seeks to contribute to a better understanding of this distinct cohort of service users and in turn to inform more equitable and culturally responsive service provision not only in Ireland but also for other marginalized communities internationally.

## 2 MATERIALS AND METHODS

### 2.1 Study design

This retrospective cross‐sectional study examined attendances of female patients who identified as members of the Irish Traveller community at the national SATU network between 2017 and 2023, comparing them with all other female attenders. The study included all female attendances at SATU during the study period. An attendance was defined as the first presentation to a SATU following a sexual assault, either for a sexual health screening or a forensic examination. Exclusion criteria included individuals who did not identify as female, those who did not disclose a sexual assault, or those who did not disclose concern that a sexual assault may have occurred.

### 2.2 Ethical approval

Ethical approval for this study was sought and granted from the Research Advisory and Guideline Group, Rotunda Hospital, Dublin, Ireland. As the data analyzed were irrevocably anonymized, individual patient consent was not required.

### 2.3 Study setting and population

In the Republic of Ireland, the SATU network comprises six units providing 24/7 care for individuals of all genders aged 14 years and older who disclose sexual assault.^10^ SATUs address medical, psychological, and emotional needs while also providing forensic examination to aid criminal investigation. Forensic examinations are conducted by specially trained doctors or nurses, with patients choosing between police involvement, health‐check only (sexual health screening), or forensic examination without immediate police involvement (evidence storage).

After each attendance, anonymized details are entered from the (paper) medical chart into a national database. The data that are recorded on this database include demographic details of the patient, incident (e.g., assailant‐victim relationship, location, time of incident) and attendance details (e.g., type of attendance, day/time of attendance, time from incident to attendance).

### 2.4 Study protocol and data analysis

Irrecoverably anonymized patient demographic data as well as incident and attendance details were imported into Microsoft Excel from the national SATU database. The data were then coded and imported into SPSS (version 31; IBM, Armonk, NY, USA). Descriptive bivariate analysis was performed to study associations between various characteristics of the assault and attendance details. We report numbers and percentages for descriptive statistics. The *χ* ^2^‐test was performed to compare relative frequencies. Odds ratios (OR) and 95% confidence intervals (CI) were also calculated; statistical significance was defined as a *P* value less than 0.05, describing the probability that an association was due to chance.

## 3 RESULTS

During the study period there were 5942 female attendances at the SATU network where a sexual assault or a concern for sexual assault was disclosed. Of these, 54 (0.9%) women identified as Irish Travellers. The number of attendances per year is shown in Figure 1. There was an increase in numbers during 2020 and 2021, which coincides with the global COVID‐19 pandemic.

**FIGURE 1:**
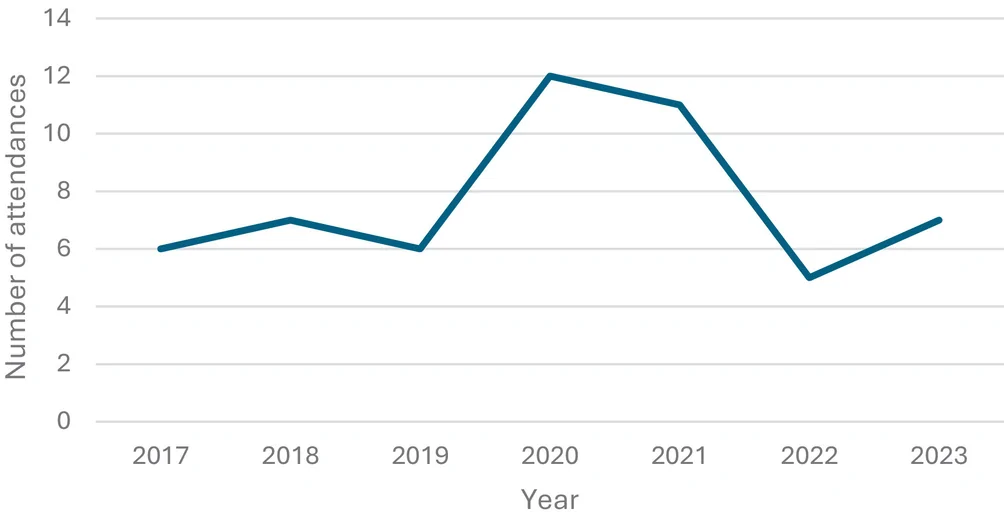
Number of female Irish Traveller attendances per year to the Sexual Assault Treatment Unit network.

The average age of attenders was 25.92 ± 11.45 years; the age groups of attenders are depicted in Figure 2. As regards employment, the majority were listed as unemployed (*n* = 30), with 14 being in full‐time education (school or third‐level education), with others being listed as being employed or retired (*n* = 10).

**FIGURE 2:**
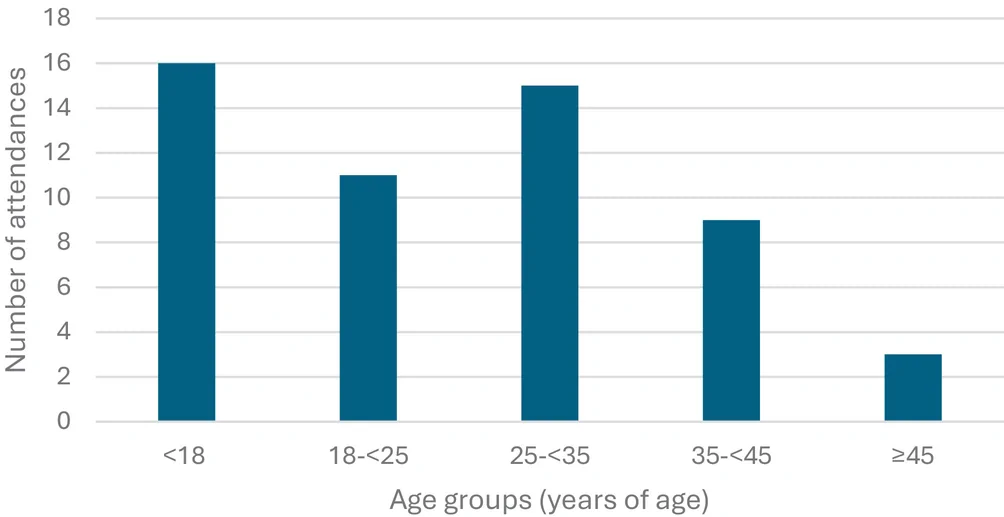
Age groups of female Irish Traveller attendances to the Sexual Assault Treatment Unit network.

Regarding their attendance at the SATU, 32 attended for a forensic examination with involvement of the Gardai (Irish police force), eight attended with the Gardai with no forensic examination performed (e.g., outside the forensic sampling time‐line/did not consent for forensic examination), two attended for a forensic examination without Garda involvement, and 12 attended for a health‐check or advice. The majority attended during the day (41, 75.9%). Most of the victims attended within 7 days of the assault (35, 64.8%), but six attended (11.1%) between more than 7 days but less than 1 month after the assault. Six (11.1%) disclosed long‐term abuse or forced prostitution and seven (13%) attended more than a month after the assault.

In relation to the assault, the victim‐assailant relationships are shown in Table 1.

**TABLE 1:** Description of relationship of victim to assailant^a^.

Relationship	Cases (*n* = 54)
Acquaintance <24 h	7 (12.9)
Acquaintance >24 h	8 (14.8)
Friend	4 (7.4)
Intimate partner	5 (9.3)
Ex‐intimate partner	7 (12.9)
Family	13 (24.1)
Stranger	9 (16.7)
Unknown	1 (1.9)

^a^ Data are presented as number (percentage).

The location of the assault was disclosed as being outdoors (field/park/other) in 13 (24.1%) cases, at the victim's home in 14 (25.9%) cases, in the assailant's home in seven (12%) cases, and in a vehicle in seven (13%) cases; eight (14.8%) cases were described as ‘other indoors’ and the remainder were unsure or unknown (9.3%, *n* = 5). Multiple perpetrator sexual assaults accounted for four cases.

Use of a weapon was disclosed in 13 (24.1%) cases, bodily restraint in 18 (33.3%) cases, and verbal threats were described in 22 (40.7%) cases.

Alcohol consumption by the victim in the 24 h before the assault was disclosed in 29 (53.7%) cases, with drug use being disclosed in six (11.1%) cases.

Irish Traveller women were significantly less likely to attend within the first 24 h following an incident when compared with other female attenders (24.1% vs 39.7%; OR 0.48, 95% CI 0.26–0.90, *P* = 0.02).

The assailant was significantly more likely to be a family member (24.1% vs 3.7%; OR 8.25, 95% CI 4.36–15.61, *P* < 0.001) or an intimate or former intimate partner (22.2% vs 12.3%; OR 2.04, 95% CI 1.07–3.89, *P* = 0.03) among Irish Traveller women compared with other female attenders. Irish Traveller women were significantly less likely to have consumed alcohol in the 24 h before the assault (46.3% vs 72.2%; OR 0.33, 95% CI 0.19–0.57, *P* < 0.001).

## 4 DISCUSSION

This study represents the first examination of Irish Traveller women attending the national SATU network following sexual assault and is likely one of the first papers detailing sexual violence within this population. Although Irish Traveller women accounted for a small proportion of overall attendances (0.9%), this must be interpreted within the broader context of systemic marginalization, underreporting of sexual violence, and documented barriers to service engagement among Irish Traveller communities. Our findings highlight several important differences from the overall female SATU population, including a significantly higher likelihood of assaults perpetrated by family members or intimate/former intimate partners, lower reported alcohol consumption before the assault, and delayed presentation greater than 24 h. These patterns suggest that sexual violence experienced by Irish Traveller women may be more likely to occur within known or familial contexts and may be shaped by complex social, cultural, and structural factors influencing disclosure and healthcare‐seeking behaviors.

First, it is likely that this number represents only a small minority of women within the Irish Traveller community that have been sexually assaulted. There are several reasons as to why people do not seek help after sexual assault; however, there are unique challenges that marginalized groups experience when accessing healthcare.^4^, ^11^ These include fear of stigma, concern about community repercussions, distrust of authorities, or previous negative healthcare experiences.^12^ It is important to note therefore that service utilization data should not be interpreted as a proxy for community prevalence.

Irish Traveller women in this study were significantly more likely to report assaults perpetrated by family members or intimate/former intimate partners compared with other female attenders. This finding suggests that sexual violence within this cohort may more frequently occur within close relational contexts rather than as stranger assaults. Abuse within familial or intimate networks presents unique challenges, including heightened barriers to disclosure, fear of social exclusion, economic dependence, and the risk of ongoing coercion or retaliation.^5^, ^12^, ^13^ In close‐knit communities, concerns about reputation, honor, and kinship dynamics may further complicate reporting.^14^, ^15^ These findings underscore the importance of recognizing sexual violence within domestic and familial settings and ensuring that services are equipped to manage complex safeguarding scenarios that may arise.

Another interesting finding was that the number of Irish Traveller women that attended during the years of the COVID‐19 pandemic increased despite the overall number of attendances at the SATU network reducing over that timeframe.^16^ This is also in the context of an increase in intimate partner violence/family member sexual assault attendances during that same time period, both in Ireland and elsewhere.^16^, ^17^ This highlights that while widespread government‐imposed restrictions may have reduced the amount of sexual violence perpetrated by strangers, these same restrictions unfortunately heightened the risk of domestic incidents perpetrated by family members and intimate partners.

As regards the timing of presentation, Irish Traveller women were significantly less likely to attend a SATU within 24 h of assault. Early presentation is clinically important for forensic evidence collection, emergency contraception, and prophylaxis against sexually transmitted infections.^18^ Delayed presentation may reflect structural barriers such as geographic isolation, limited transport access, and socioeconomic disadvantage.^19^ It may also reflect psychosocial factors including shame, fear of community repercussions, uncertainty about engaging with the criminal justice system, or lack of awareness of available services.^20^, ^21^ These delays highlight the need for targeted community outreach and culturally appropriate information campaigns to facilitate timely access to care. Ongoing engagement with Pavee Point, a national non‐governmental organization comprising Irish Travellers, Roma, and non‐Travellers/Roma that works at local, regional, national, and international levels to address Irish Traveller and Roma inequalities and promote human rights, and with other stakeholders in the health service that support social inclusion may be of significant benefit.

The present study also found that Irish Traveller women were significantly less likely than other female attenders to report consuming alcohol in the 24 h before the assault. Previous research has shown that alcohol use is not typically elevated in cases of intimate partner violence, suggesting that different forms of violence may follow distinct pathways.^22^ This aligns with our findings, as a large proportion of assaults involving Irish Traveller women occurred within familial or intimate partner contexts—settings that are generally less associated with alcohol use by the victim. This finding also challenges the societal narrative that sexual violence is primarily linked to intoxication in social settings.

These findings highlight the importance of ensuring that sexual assault services are responsive to the needs of Irish Traveller women. Building cultural awareness among staff, addressing discrimination within healthcare settings, and working in partnership with Irish Traveller organizations may help to strengthen trust and improve access to care. Practical steps such as targeted outreach, clear and accessible information about services, and stronger referral pathways could further support engagement.

A key strength of this study is the use of a national dataset spanning six SATUs over a 7‐year period. To our knowledge, this is the first published analysis focusing specifically on Irish Traveller women attending sexual assault services in Ireland. The inclusion of a comparator group of other female attenders allows for a more meaningful interpretation of the differences observed and adds important context to the findings. In doing so, this study begins to address a clear gap in the literature and provides an initial evidence base in an area where research has been notably limited.

There are, however, important limitations to consider. The relatively small number of Irish Traveller attendances limits the statistical power of the analysis and restricts the extent to which the findings can be interpreted and generalized. As a retrospective study, the analysis depended on routinely collected clinical data, which may be incomplete or inconsistently recorded. Irish Traveller ethnicity was self‐identified and may not have been disclosed in all cases, meaning some attendances may not have been captured. Finally, these findings reflect patterns of service use rather than prevalence in the wider community. As such, they cannot be used to estimate the overall burden of sexual violence among Irish Traveller women in Ireland.

In conclusion, the present study provides the first national analysis of Irish Traveller women attending SATUs in Ireland and highlights distinct patterns in perpetrator relationship and timing of presentation compared with other female attenders. The findings suggest that sexual violence experienced by Irish Traveller women more frequently occurs within familial and intimate relationships and that barriers to early presentation may exist. Although the small sample size limits generalizability, the study identifies important considerations for culturally responsive and equity‐focused service provision. Further research, particularly community‐engaged and qualitative work, is needed to better understand the broader prevalence, context, and support needs of Irish Traveller women and other ethnic minorities affected by sexual violence.

## AUTHOR CONTRIBUTIONS

DK, ME, KF, and AMcE contributed to the conception and design of the study; DK, CP, KOH, and NM contributed to the acquisition and analysis of data; DK, CP, KOH, KF, LF, AMcE, ME, and NM contributed to the drafting or critical revision of the manuscript and approved the final version. All authors agree to be accountable for all aspects of the work.

## CONFLICT OF INTEREST STATEMENT

Professor Maeve Eogan was a guest editor for the special edition on DSGBV; however, she was not part of the peer‐review process. The other authors have no conflicts of interest.

## Data Availability

Research data are not shared.
